# Supplementing Garlic Nanohydrogel Optimized Growth, Gastrointestinal Integrity and Economics and Ameliorated Necrotic Enteritis in Broiler Chickens Using a *Clostridium perfringens* Challenge Model

**DOI:** 10.3390/ani11072027

**Published:** 2021-07-07

**Authors:** Doaa Ibrahim, Tamer Ahmed Ismail, Eman Khalifa, Shaimaa A. Abd El-Kader, Dalia Ibrahim Mohamed, Dalia T. Mohamed, Sara E. Shahin, Marwa I. Abd El-Hamid

**Affiliations:** 1 Department of Nutrition and Clinical Nutrition, Faculty of Veterinary Medicine, Zagazig University, Zagazig 44519, Egypt; 2 Department of Clinical Laboratory Sciences, Turabah University College, Taif University, P.O. Box 11099, Taif 21944, Saudi Arabia; t.ismail@tu.edu.sa; 3 Department of Microbiology, Faculty of Veterinary Medicine, Matrouh University, Matrouh 51511, Egypt; khalifa.eman@alexu.edu.eg; 4 Department of Bacteriology, Zagazig Branch, Agriculture Research Center, Animal Health Research Institute, Zagazig 44519, Egypt; Dr.shaimaahany@gmail.com; 5 Department of Biochemistry, Zagazig Branch, Agriculture Research Center, Animal Health Research Institute, Zagazig 44519, Egypt; Daliaibrahim79@yahoo.com; 6 Department of Pathology and Clinical Pathology, Zagazig Branch, Agriculture Research Center, Animal Health Research Institute, Zagazig 44519, Egypt; Daliatalaat38@gmail.com; 7 Department of Animal Wealth Development, Biostatistics, Faculty of Veterinary Medicine, Zagazig University, Zagazig 44519, Egypt; saraesam2011@gmail.com; 8 Department of Microbiology, Faculty of Veterinary Medicine, Zagazig University, Zagazig 44519, Egypt

**Keywords:** broilers, garlic nano-hydrogel, growth, clostridium perfringens, intestinal barriers, real-time PCR

## Abstract

**Simple Summary:**

Necrotic enteritis (NE) is one of the most serious diseases in terms of economic losses. Aspects related to application of nanotechnology to control outbreaks of NE due to *Clostridium perfringens* (*C. perfringens*) are not completely understood. Hence, the purpose of this study was to evaluate the beneficial effects of garlic nano-hydrogel (G-NHG) on the performance, intestinal integrity, economic returns and alleviation of the intestinal *C. perfringens* levels using an NE challenge model in broiler chickens. Dietary inclusion of 400 mg/kg of G-NHG improved body weight gain (BWG) and feed conversion ratio (FCR). The digestive enzymes and tight junction and gut barrier-related genes expression were negatively impacted post *C. perfringens* challenge, resulting in a decrease in BWG with an increase in FCR. Meanwhile, G-NHG supplementation decreased *C. perfringens* levels, mortality rates and intestinal lesion score, and thereby improved intestinal permeability measurements, which consequently resulted in improved growth performance parameters. In conclusion, G-NHG markedly ameliorated the negative effects of *C. perfringens* challenge, which positively reflected on the growth performance of challenged birds, suggesting its beneficial effects in controlling *C. perfringens* infection in broiler chickens.

**Abstract:**

Necrotic enteritis (NE) caused by *Clostridium perfringens* (*C. perfringens)* results in impaired bird growth performance and increased production costs. Nanotechnology application in the poultry industry to control NE outbreaks is still not completely clarified. Therefore, the efficacy of dietary garlic nano-hydrogel (G-NHG) on broilers growth performance, intestinal integrity, economic returns and its potency to alleviate *C. perfringens* levels using NE challenge model were addressed. A total of 1200 male broiler chicks (Ross 308) were assigned into six groups; four supplemented with 100, 200, 300 or 400 mg of G-NHG/kg diet and co-challenged with *C. perfringens* at 21, 22 and 23 d of age and two control groups fed basal diet with or without *C. perfringens* challenge. Over the total growing period, the 400 mg/kg G-NHG group had the most improved body weight gain and feed conversion efficiency regardless of challenge. Parallel with these results, the mRNA expression of genes encoding digestive enzymes (alpha 2A amylase (AMY2A), pancreatic lipase (PNLIP) and cholecystokinin (CCK)) and intestinal barriers (junctional adhesion molecule-2 (JAM-2), occludin and mucin-2 (Muc-2)) were increased in groups fed G-NHG at higher levels to be nearly similar to those in the unchallenged group. At 14 d post challenge, real-time PCR results revealed that inclusion of G-NHG led to a dose-dependently decrease in the *C. perfringens* population, thereby decreasing the birds’ intestinal lesion score and mortality rates. Using 400 mg/kg of G-NHG remarkably ameliorated the adverse effects of NE caused by *C. perfringens* challenge, which contributed to better growth performance of challenged birds with rational economic benefits.

## 1. Introduction

Gut health encompasses a variety of physiological and functional features essential for a cost-effective and higher growth rate, especially in intensive poultry production systems. The small intestine is not only the principal site for regulating an animal’s digestion and absorption, it also tightly regulates the passage of pro-inflammatory molecules, microorganisms and toxins to act as a barrier against pathogens and toxins. Bacterial infection is among the main causative agents [1,2] that adversely affects the intestinal health and impairs the productive performance and immune status of poultry. Necrotic enteritis (NE), especially necro-hemorrhagic enteritis, is one of the most infectious diseases that threatens the poultry industry [3], resulting in a great economic burden in terms of treatment costs and estimated productivity losses of 6 billion US dollars worldwide [4]. It is a result of over proliferation of *Clostridium perfringens* (*C. perfringens*), which represents the most common member of the normal intestinal microbiota of poultry. It is an anaerobic Gram-positive, spore-forming bacterium that is grouped into five types of toxins (A, B, C, D and E) based upon the production of four main toxinotypes (alpha, beta, epsilon and iota) with *C. perfringens* type A being the major causative agent of hemorrhagic NE [5]. Based on *C. perfringens* expansion of the toxin-based typing scheme, two new toxinotypes have been established (*C. perfringens* type F producing *C. perfringens* enterotoxin, but not beta, epsilon or iota toxins and *C. perfringens* type G producing NetB toxin) [6]. The principles are described, as is a mechanism by which new toxinotypes can be proposed and subsequently approved. these criteria consist of isolates that produce Type F strains will include strains responsible for *C. perfringens*-mediated human food poisoning and antibiotic associated diarrhea. Its growth rate is greatly affected by the temperature; it grows rapidly at elevated temperatures with a generation time of 8 to 10 min [7]. The pathogenesis of *C. perfringens* infection can be broken down into a number of stages including colonization of the host tissues, nutrients acquisition to permit further multiplication, evasion of the host immune defenses and transmission of toxins causing host damage [8]. *C. perfringens* α-toxin suppresses innate immunity via neutrophil differentiation inhibition, which explains how pathogenic *C. perfringens* can evade the host immune system [9]. Additionally, potent inflammatory reactions caused by *C. perfringens* infection may impair nutrient digestion and absorption, and thus, suppress the chicken’s growth rate [10,11,12,13]. Collectively, many studies have indicated that inhibition of pathogenic bacteria and reinforcement of the intestinal barrier integrity may be closely interlinked [14].

Banning of antibiotic growth promoters (AGPs) used to control NE has forced the poultry industry to develop natural alternatives for improving the growth performance and maximizing the production efficiency [15]. Thus, essential oils (EOs) are generally recognized as safe and effective promising natural alternatives with growth promoting activities [16] and antibacterial properties against a wide range of pathogenic bacteria [17]. Furthermore, EOs have been described to strengthen the chicken’s mucosal barriers and intestinal integrity [18,19] and modulate the expression of immune related genes [20]. Garlic and garlic-related products, such as garlic oils, essential oils, aged garlic extracts and so forth, provide several beneficial effects for human and animal health. They have been reported to exert antioxidant, antimicrobial, antiviral, antifungal, hypercholesteremic and immuostimulating properties [21]. Moreover, they have positive effects on the digestion of broiler chickens due to its very rich aromatic essential contents [22,23]. Interestingly, the beneficial single and combined effects of garlic essential oil and oregano in reducing the ileal clostridia counts in broiler chickens were documented [24]. However, the instability and volatility of garlic bioactive compounds may limit its application in poultry farms. Therefore, searching for new techniques that can enhance garlic stability and preservation is a major concern for feed manufacturers to maximize its efficacy and application. Nanotechnology is one of the most innovative and promising strategies used to protect bioactive constituents against oxidation, heat or volatilization, maintain delivery, uniform size distribution and storage stability, mask undesirable flavors and increase the shelf life without altering their physical, chemical and functional properties [25]. The incorporation of plants extracts into nanocomposite hydrogels can enhance their activity at low doses when used in poultry production [26]. Nanotechnology application in poultry production system is still in its nascent stage. Despite this interest, there have been no reports to the best of our knowledge on the use of nanoparticles (NPs) from garlic essential oil in poultry farming. Therefore, the current study was undertaken to explore, for the first time, the efficacy of garlic nano-hydrogel (G-NHG) on broilers growth performance, intestinal integrity and net economic returns. Moreover, in this study, we adopted a *C. perfringens* challenge model to investigate the potency of G-NHG on the intestinal proliferation of *C. perfringens*.

## 2. Materials and Methods

### 2.1. Garlic Nano-Hydrogel Preparation

**Figure 1:**
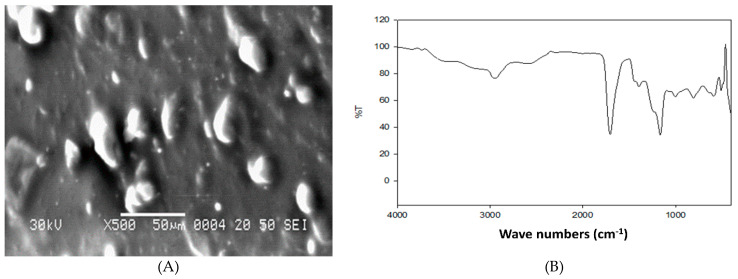
Scanning electron microscopy (**A**) and Fourier-transform infrared spectroscopy (FTIR, **B**) of garlic nano-hydrogel (G-NHG).

Sodium alginate (A-2033), garlic oil (W250320) and acrylic acid (147230) were purchased from Sigma-Aldrich (Saint Louis, MO, USA). Sodium alginate was dissolved in 1% distilled water using various ratios of acrylic acid. Network structure of sodium alginate was formed by gamma irradiation using hydrogels solutions, and then acrylic acid and sodium alginate solutions were blended until complete compatibility. The prepared mixture was then subjected for gamma irradiation (35 kGy) at the Atomic Energy Authority, Egypt. Characterization of the prepared G-NHG was done using a scanning electron microscope (Figure 1A) and Fourier-transform infrared spectroscopy (FTIR, Figure 1B).

### 2.2. Birds, Diets and Experimental Design

Approval for the experimental protocols was obtained from the Local Animal Ethics Committee of Zagazig University, Egypt (Approval No. ZU-IACUC/F/141/2020). A total of 1200 one-day old male broiler chickens (Ross 308), purchased from a local commercial hatchery, were used for a 38-day experiment. Chicks were individually weighed and randomly assigned into six groups (200 birds/group) and each group involved 20 replicates with 10 birds each. Birds in the negative control (NC) group were unchallenged, but birds in the positive control (PC) group were challenged with *C. perfringens*. Both control groups were fed the basal diet without G-NHG supplementation. Correspondingly, birds in the four treated groups were fed the basal diet containing 100, 200, 300 or 400 mg/kg diet of G-NHG [27] and challenged with *C. perfringens* at 21, 22 and 23 d of age. Broilers were fed mash form diets for starter (d 1–10), grower (d 11–20) and finisher (d 21–38) periods, with free access to feed and drinking water. The antibiotic- and coccidiostat-free balanced commercial diets were formulated to meet the recommendations of Ross broiler nutrition specifications [28], as shown in Table 1. Chemical analysis of various diets was performed in line with the Association of Official Analytical Chemists, AOAC [29].

### 2.3. Monitoring Chicken’s Growth Performance

The average daily feed intake (FI) and body weight (BW) were determined to calculate the body weight gain (BWG) and feed conversion ratio (FCR) at the end of each period [30]. The FI, BWG and FCR were calculated for the entire experimental period (d 1–38) as previously reported by Ibrahim et al. [31,32].

### 2.4. Clostridium Perfringens Challenge Model

A coccidia-free *C. perfringens* challenge model was applied in this study as previously described by Yin et al. [33]. *Clostridium perfringens* type A strain was freshly isolated from a field avian case suffering from NE in a commercial broiler chicken flock at Sharkia Governorate, and it was able to induce the enteric disorders and severe lesions typical for NE. The strain was cultivated anaerobically on tryptose sulphite cycloserine agar (Oxoid, Basingstoke, UK) plates at 37 °C for 18 h. The black colonies of *C. perfringens* were confirmed by conventional and molecular methods. The strain was further confirmed to be of type A by PCR amplification of the alpha toxin gene. The actual *C. perfringens* concentration in the prepared challenge inoculum was approximately 1.0 × 10^8^ colony forming units (CFU)/mL.

On d 21, 22 and 23 of the experiment, all birds in PC and G-NHG treated groups were individually challenged twice a day with freshly prepared inoculum of *C. perfringens* utilizing a crop gavage, whereas birds in the NC group were treated orally gavaged with 1 mL of physiological saline solution. Establishment of *C. perfringens* infection in birds displaying NE typical gross lesions was confirmed by detection of *C. perfringes* and their alpha toxin gene by culture and PCR-based methods, respectively, as mentioned above. The birds were frequently observed for any clinical signs of NE and the mortality was registered daily. Birds that died or culled due to unhealthy conditions were necropsied and scored for the gross lesions of NE according to earlier established criteria [34]. Before necropsy, the birds were anesthetized via intraperitoneal injection of sodium pentobarbital (40 mg/kg) and euthanized by cervical dislocation according to the American Veterinary Medical Association guidelines for the euthanasia of animals [35].

### 2.5. Expression Analysis by Reverse Transcription Quantitative Real-Time PCR (RT-qPCR)

Pancreatic and duodenal mucosal samples were collected at the end of the experiment (38th day of age) for determining the expression levels of genes encoding the digestive enzymes (alpha 2A amylase (AMY2A), pancreatic lipase (PNLIP) and cholecystokinin (CCK)) and three molecules related to the intestinal barrier (junctional adhesion molecule-2 (JAM-2), occludin and mucin-2 (Muc-2)). Total RNA was then isolated using QIAamp RNeasy Mini kit (Qiagen, Hilden, Germany) as recommended by the manufacturer. The concentration of the eluted RNA was determined spectrophotometrically at 260 nm and the RNA purity was then assessed by calculating the ratio of absorbance values at 260 and 280 nm. One-step RT-qPCR assay was performed on the Stratagene MX3005P real-time PCR detection system using a commercial QuantiTect SYBR Green RT-PCR Kit (Qiagen, Hilden, Germany). All PCR measurements were applied in triplicate. The specificity of each PCR amplification assay was verified using a final melting curve analysis. The levels of different transcripts were then normalized to the expression of glyceraldehyde 3-phosphate dehydrogenase (GAPDH) as an endogenous control. All gene-specific primer sequences utilized in RT-qPCR assay are listed in Table 2. The results of relative mRNA expression of investigated genes were evaluated using the 2^−ΔΔCt^ method [36].

### 2.6. Biochemical Indices

Blood samples were aseptically obtained from the wing vein of birds (one bird/replicate) for immunological assays and biochemical analysis. Serum was separated by centrifuging the blood samples for 15 min at 2000 rpm and then it was stored at −20 °C for various analysis. At 30 and 38 d of age (7 and 14 d post challenge, respectively), estimation of lysozyme, nitric oxide (NO) and myeloperoxidase (MPO) activities were conducted using commercial kits (Jiancheng Biotechnology Institute, Nanjing, China). For determining the biochemical parameters at the end of experimental period, the concentrations of alanine transaminase (ALT), aspartate transaminase (AST), creatinine, uric acid, cholesterol, triglycerides total, very low-density lipoprotein (VLDL), low density lipoprotein (LDL) and high-density lipoprotein (HDL) were estimated using standard kits (Span Diagnostic Ltd., Sachin, India).

### 2.7. Intestinal Lesion Score

On d 30 and 38, five birds/replicate were randomly picked out, slaughtered and sacrificed. The small intestine from each bird was aseptically removed and subjected to lesion scoring using a scale from 0 to 4 as follows: 0 = no apparent lesions (normal intestinal appearance); 0.5 = extremely congested serosa and mesentery distended with blood; 1 = friable and thin walled small intestine; 2 = focal necrotic lesions, ulceration and/or gas production; 3 = gas-filled and hemorrhagic intestines and patches of necrosis and 4 = severe and diffused mucosal necrosis typical of the field cases [34]. Birds that exhibited lesion scores of 2 or more were considered to be NE positive.

### 2.8. Quantitative Detection of Ileal C. perfringens by Real-Time PCR

On d 30 and 38, absolute quantitation of *C. perfringens* populations was carried out by quantitative real-time PCR (RT-PCR) assay. Genomic DNA was extracted from ileal digesta using the commercial QIAamp DNA stool kit (Qiagen, Hilden, Germany) according to the manufacturer’s recommendations. Eluted DNA concentration was assessed with a NanoDrop^TM^ 2000 spectrophotometer (Thermo Fisher Scientific Inc., Waltham, MA, USA). After that, purified DNA samples were stored at −80 °C until analysis. The ileal *C. perfringens* numbers were enumerated, in duplicate, by SYBR-Green-I based RT-PCR assay performed on the Stratagene MX3005P RT-PCR machine using SYBR Premix Ex Taq™ kit (TaKaRa, Maebashi, Japan) following the manufacturer’s protocol. The primer pair; CPerf165F: 5′-CGCATAACGTTGAAAGATGG-3′ and CPerf269R: 5′-CCTTGGTAGGCCGTTACCC-3′ (Invitrogen, Mulgrave, VIC, Australia) targeting the 16S rRNA gene of *C. perfringens* was used [37]. Ten-fold serial dilutions of DNA extracted from pure *C. perfringens* cultures were done on the 96-well plate to generate a standard curve for RT-PCR. The concentration of *C. perfringens* in each sample was measured in terms of log^10^ CFU per gram of the ileal digesta.

### 2.9. Economic Analysis

Cost return analysis was performed to evaluate the economic advantage of supplementing broilers diet with varying levels of G-NHG. According to Ibrahim et al. [31], the feed costs (average variable costs) were calculated by multiplying the actual FI for the whole feeding period with the prevailing prices. The total revenue (TR) = live body weight × price/kg. The net profit (NP) was calculated by subtracting total costs (TC) from TR. Feed costs/kg weight gain = feed conversion × cost of one kg diet. The economic efficiency was calculated from the input/output analysis as per the prevailing market price of the experimental diets at the time of the experiment. Economic efficiency (EE) = NP/feed costs. Profitability index (PI) = NP/TR. Moreover, the mortality losses were estimated as previously documented by Williams and Pant et al. [38,39]:

Cost of mortality = no. of died bird × (average fixed cost + average cost of bird that reared until death)(1)

Profit loss = (no. of dead birds x TR) − (no. of dead birds x total costs of bird that reared until death)(2)

The sum of Equations (1) and (2) implies the mortality losses.

### 2.10. Statistical Analysis

Statistical analysis was performed using the General linear method (GLM) method of SPSS. The homogeneity among treated groups was performed using the Levene test and normality was evaluated using the Shapiro–Wilk test. Tukey’s test was carried out to separate the mean values when the differences were significant. Data variation was expressed as standard error of the mean (SEM) and the statistical significance was set at a *P* value less than 0.05 (typically ≤ 0.05). The experimental unit was a pen of broilers. All graphs were made by GraphPad Prism software Version 8 (San Diego, CA, USA).

## 3. Results

### 3.1. Growth Performance Parameters 

The results concerning growth performance parameters of broiler chickens at starter, grower (preinfection) and finisher periods (postinfection) are presented in Table 3. During starter period, the BWG of broiler chickens was significantly (*p* < 0.05) increased in all groups supplemented with G-NHG, except for the group fed 100 mg/kg of G-NHG. Moreover, the FI during this period was significantly (*p* < 0.05) decreased in the 400 mg/kg G-NHG-supplemented group, while it was not affected by dietary G-NHG in other groups. Broilers fed 400 mg/kg of G-NHG showed improved FCR when compared with other experimental groups. During the grower period, all experimental birds fed G-NHG at different levels showed increased BWG. Additionally, groups fed 300 and 400 mg/kg of G-NHG exhibited the most improved FCR. During the finisher period, the BWG of broiler chickens fed 400 mg/kg of G-NHG was not suppressed by *C. perfringens* challenge. Moreover, other groups supplemented with G-NHG showed a significant (*p* < 0.05) increase in BWG when compared with the PC group. Furthermore, FCR was enhanced in groups supplemented with higher levels of G-NHG unlike the PC group. At the end of experimental period, *C. perfringens* challenge caused significant growth retardation in all experimental groups, except for the 400 mg/kg G-NHG-supplemented group, which displayed increased final BWG regardless of *C. perfringens* challenge. Moreover, groups supplemented with 200 and 300 mg/kg of G-NHG exhibited a significant (*p* < 0.05) increase in the final BWG when compared with the PC group. Overall, during the growing period, FCR was not affected by *C. perfringens* challenge in birds fed 400 mg/kg of G-NHG. Additionally, FCR of birds that received 200 and 300 mg/kg of G-NHG were affected by *C. perfringens* challenge, but it was not impaired as in the PC group.

### 3.2. Gene Expression Analysis

#### 3.2.1. Digestive Enzymes

**Figure 2:**
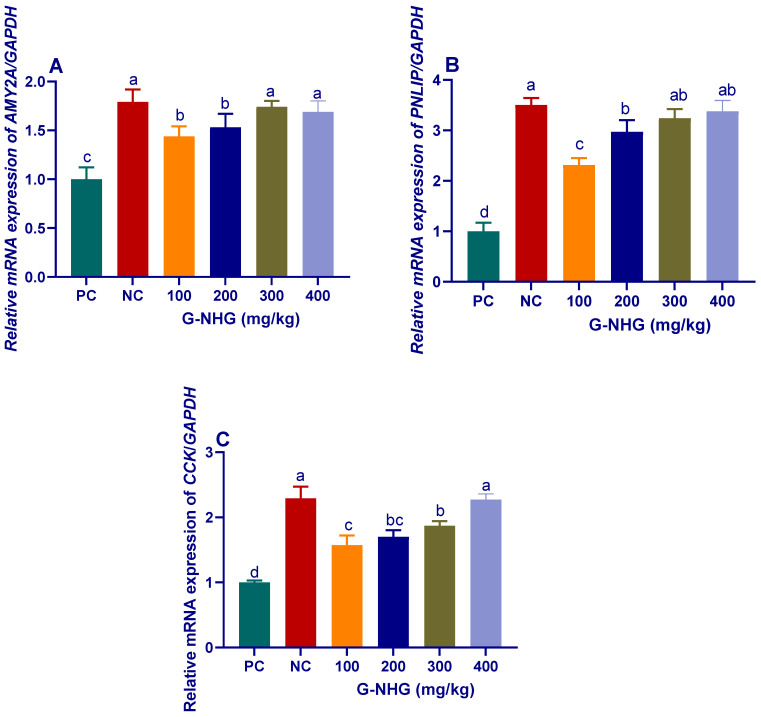
Levels of alpha 2A amylase (*AMY2A*, **A**), pancreatic lipase (*PNLIP*, **B**) and cholecystokinin (*CCK*, **C**) mRNA expression in broiler chickens fed different levels of G-NHG and challenged with *C. perfringens* on d 21, 22 and 23 of the experiment as measured by RT-qPCR assay. Values are means with their SE in bars. PC (positive control): birds fed a basal diet and challenged with *C. perfringens*, NC (negative control): birds fed a basal diet, G-NHG (100, 200, 300 or 400 mg/kg)-treated groups: birds fed a basal diet supplemented with 100, 200, 300 or 400 mg/kg of garlic nanohydrogel and challenged with *C. perfringens*. ^a–d^ Means within the same column carrying different superscripts are significantly different at *p* < 0.05.

At the end of feeding trail, the mRNA expression of AMY2A, PNLIP and CCK genes were increased in response to dietary inclusion of G-NHG when compared to PC group (Figure 2). The highest upregulation of AMY2A was observed in groups received 300 and 400 mg/kg of G-NHG regardless to *C. perfringens* challenge (Figure 2A). Additionally, both PNLIP (Figure 2B) and CCK (Figure 2C) genes were upregulated in groups that received G-NHG in a dose-dependent manner. Regardless of *C. perfringens* challenge, dietary inclusion of G-NHG at higher levels maintained the upregulated levels of genes encoding digestive enzymes to be nearly the same as those in the NC group.

#### 3.2.2. Tight Junction Proteins and Mucin-2

**Figure 3:**
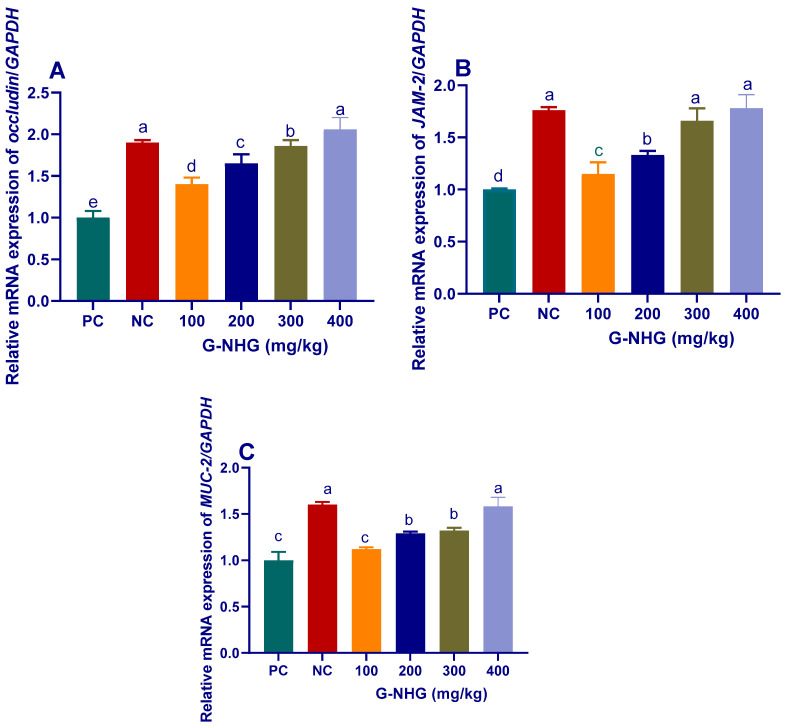
Transcript levels of occludin (**A**), junction adhesion molecules-2 (*JAM-2*, **B**) and mucin-2 (*Muc-2*, **C**) genes in the duodenal mucosal samples of broiler chickens fed different levels of G-NHG and challenged with *C. perfringens* on d 21, 22 and 23 of the experiment as measured by RT-qPCR assay. Values are means with their SE in bars. PC (positive control): birds fed a basal diet and challenged with *C. perfringens,* NC (negative control): birds fed a basal diet, G-NHG (100, 200, 300 or 400 mg/kg)-treated groups: birds fed a basal diet supplemented with 100, 200, 300 or 400 mg/kg of garlic nanohydrogel and challenged with *C. perfringens*. ^a–e^ Means within the same column carrying different superscripts are significantly different at *p* < 0.05.

The transcriptional levels of genes encoding tight junction proteins, TJP (occludin and JAM-2), were significantly (*p* < 0.05) increased in groups received G-NHG regardless to challenge with *C. perfringens.* Meanwhile, all groups except that received 100 mg/kg of G-NHG showed a significant (*p* < 0.05) upregulation of Muc-2 gene (Figure 3). The expression of occludin was significantly (*p* < 0.05) increased with increasing levels of G-NHG (up to 2.06-fold) when compared with PC group (Figure 3A). Groups received 300 and 400 mg/kg of G-NHG showed the most significant (*p* < 0.05) upregulation of JAM-2 gene (1.7 and 1.8-fold, respectively, Figure 3B). The highest significant (*p* < 0.05) expression of Muc-2 gene was observed in birds received 400 mg/kg of G-NHG (1.5-fold, Figure 3C).

### 3.3. Serum Activities of Lysozyme, Nitric Oxide and Myeloperoxidase

Serum activities of lysozyme, NO and MPO were significantly (*p* < 0.05) increased in all groups challenged with *C. perfringens* at 7 and 14 d post challenge, unlike the NC group (Table 4). At both intervals, serum activities of lysozyme and MPO were significantly decreased (*p* < 0.05) in all groups that received G-NHG compared to the PC group. Moreover, the NO contents were reduced in birds received 300 and 400 mg/kg of G-NHG when compared with the PC group. Notably, no significant differences were detected in serum activities of MPO and NO in the NC and 400 mg/kg G-NHG-supplemented groups.

### 3.4. Serum Biochemical Parameters

At the end of the experiment, it was noticed that *C. perfringens* adversely affected the liver and kidney functions’ parameters (Table 5). Serum AST, ALT, uric acid and creatinine levels were reduced in all groups received G-NHG unlike PC group. Concerning lipid profile, serum cholesterol, triglycerides, LDL and VLDL were significantly (*p* < 0.05) decreased in all groups that received a higher dosage of G-NHG regardless of *C. perfringens* challenge; the highest level of HDL was detected in birds fed 400 mg/kg of G-NHG.

### 3.5. Intestinal Lesion Scoring

**Figure 4:**
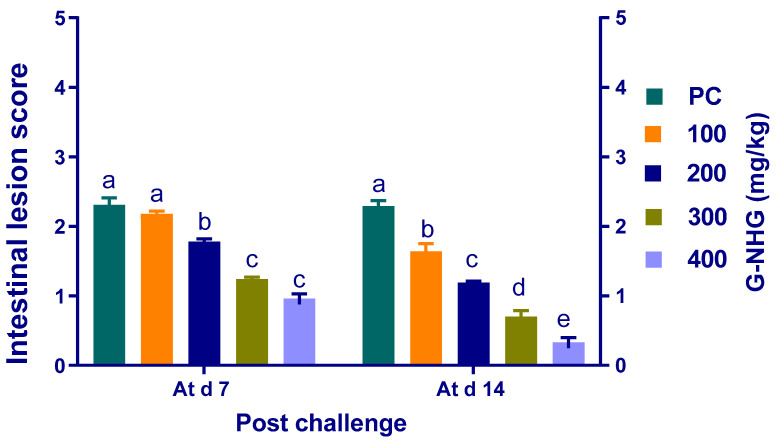
Effects of dietary supplementation with different levels of G-NHG on lesion score in the small intestine of broiler chickens at 7 and 14 d post challenge with *C. perfringens*. Values are means with their SE in bars. PC (positive control): birds fed a basal diet and challenged with *C. perfringens*, G-NHG (100, 200, 300 or 400 mg/kg)-treated groups: birds fed a basal diet supplemented with 100, 200, 300 or 400 mg/kg of garlic nanohydrogel and challenged with *C. perfringens*. ^a–e^ Means within the same column carrying different superscripts are significantly different at *p* < 0.05.

The results revealed that unchallenged birds exhibited no intestinal lesions (Figure 4). Generally, intestinal lesion scores were decreased in all groups supplemented with G-NHG in comparison with the PC group. At 7 d post challenge, the decline in intestinal scoring was significant (*p* < 0.05) in all groups that received G-NHG, except the group that received 100 mg/kg of G-NHG. Compared with PC group, intestinal lesion severity in birds fed G-NHG was significantly (*p* < 0.05) reduced in a dose-dependent manner at 14 d post challenge.

### 3.6. Quantitation of Ileal C. perfringens

**Figure 5:**
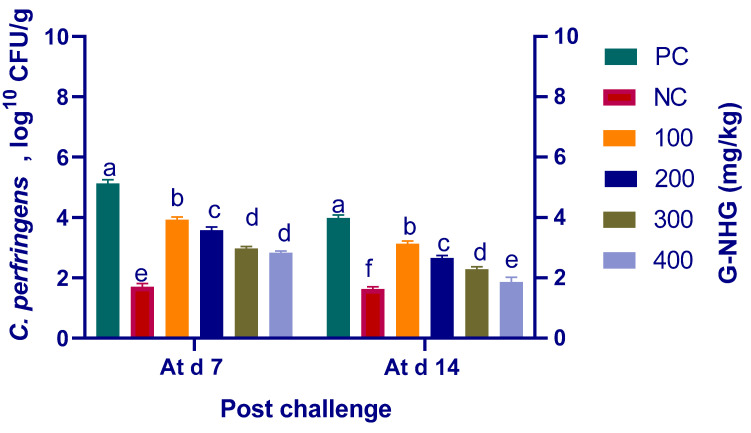
Impact of dietary supplementation of various levels of G-NHG on *C. perfringens* population in ileal digesta of broiler chickens at 7 and 14 d post challenge. Values are means with their SE in bars. PC (positive control): birds fed a basal diet and challenged with *C. perfringens,* NC (negative control): birds fed a basal diet, G-NHG (100, 200, 300 or 400 mg/kg)-treated groups: birds fed a basal diet supplemented with 100, 200, 300 or 400 mg/kg of garlic nanohydrogel and challenged with *C. perfringens*. ^a–f^ Means within the same column carrying different superscripts are significantly different at *p* < 0.05.

The results of *C. perfringens* quantification in the ileal digesta are presented in Figure 5. The results demonstrated that G-NHG at various supplementation levels significantly (*p* < 0.05) reduced *C. perfringens* log_10_ numbers of copies with respect to the PC group at 7 and 14 d post challenge. The *C. perfringens* populations decreased with increased G-NHG levels at both intervals. At 7 d post challenge, the lowest *C. perfringens* counts were observed in the birds fed 300 and 400 mg/kg of G-NHG (2.16 and 2.30 log units decreases than the PC group), but with no detectable significant differences. At 14 d post challenge, the mean values of *C. perfringens* loads corresponding to the different supplementation levels were statistically significant (*p* < 0.05). Supplementing diets with 400 mg/kg of G-NHG was found to cause a 2.14 log CFU/g reduction of *C. perfringens* ileal counts than the PC group. The mean value for *C. perfringens* populations in this group was restored near to that in the NC group.

### 3.7. Economic Evaluation Parameters

The morality rates throughout the entire rearing period ranged from 4–32% (Table 6). Use of dietary G-NHG reduced the mortality rates in birds, especially those fed 400 mg/kg of G-NHG. Herein, the highest mortality cost (*p* < 0.05) was observed in the PC group. Meanwhile, dietary G-NHG supplementation reduced mortality losses that resulted from *C. perfringens* challenge. Feed cost and total expenses increased significantly (*p* < 0.05) with increasing G-NHG levels. The group that received 400 mg/kg of G-NHG displayed the highest (*p* < 0.05) total expenses, total revenue, net profit, cost/benefit ratio, economic efficiency and profitability index irrespective of *C. perfringens* challenge. In contrast, the most negative impact on economic indicators was detected in the challenged group that did not receive G-NHG (Table 6).

## 4. Discussion

*Clostridium perfringens* infection has been evidenced to decrease feed efficiency and increase gut lesions and mortality rates, which account for higher productive losses in poultry. Using natural feed additives in the form of nanoformulation can provide great protection against *C. perfringens* infection. In the current study, we found that inclusion of G-NHG in the broiler’s diet preceding *C. perfringens* challenge could significantly improve growth performance and reduce both mortality and intestinal lesions. During the starter period, the most improved BWG and FCR were detected in the group that received 400 mg/kg of G-NHG; in the grower period, the highest BWG and superior FCR were observed in groups that received 300 and 400 mg/kg of G-NHG. During the finisher period, a similar improvement pattern was observed for BWG in 400 mg/kg G-NHG-supplemented group, with values similar to those observed in the NC group without infection. These results are in agreement with Brzóska et al. [23], who described that dietary supplementation of garlic extract at the levels of 1.50 and 2.25 mL/kg stimulated the appetite and significantly increased body gain compared to control group. Moreover, Kirubakaran et al. [40] described that garlic in broilers’ diet might increase the secretion of gastric juice, causing better digestibility and BWG. A previous study showed that supplementation of garlic to broilers’ diets improved their immune systems and digestion efficiency due to the abundance of bioactive compounds of garlic, namely, alliin, daily lsulphide and allicin [41]. Additionally, improvement in weight gain and better FCR of broiler chickens following the dietary supplementation of garlic can be attributed to allicin-active ingredients that promote the beneficial microflora in the gut, thereby improving the digestion efficiency and enhancing the energy utilization [42,43]. Peinado et al. [44] found that the compounds obtained from garlic had increased digestibility and activities of intestinal mucosal enzymes, confirming their application as alternatives to antibiotics in broiler nutrition. Moreover, higher growth rates of chickens are associated with the antimicrobial impact of garlic active ingredients on intestinal microorganisms, producing toxic metabolites and/or competing with the host for accessible nutrients.

Injured mucosa caused by *C. perfringens* results in inferior growth rate and feed conversion efficiency in poultry [45,46]. Herein, *C. perfringens* caused a retardation of BWG with an inferior FCR and high mortality rates in broiler chickens that did not receive G-NHG over the entire experimental trial. In contrast, groups received G-NHG and infected with *C. perfringens* showed an improvement in BWG and FCR in a dose-dependent manner. Interestingly, BWG and FCR of birds received 400 mg/kg of G-NHG were similar to those observed in the NC group without infection. Similarly, a previous in vivo study evaluating the effect of the dietary supplementation of garlic metabolites revealed increased BWG and higher antibody response in NE challenged chickens when compared with challenged birds with no garlic supplementation [47]. The authors concluded that the aforementioned effects were due to phenolic compounds and allicin in garlic. Moreover, rapid growth and enhanced digestion and immunity of poultry can result from the reduced expanding range of pathogens in the digestive tract upon dietary garlic supplementation [48]. Decreased BWG losses and lesion scores upon *A. Hookeri* dietary supplementation in commercial broilers experimentally challenged with NE caused by *C. perfringens* infection were reported [49]. Additionally, garlic at different concentrations lowered the cecal counts of *C. perfringens*, which have been reported to account for better performance in broiler chickens [50]. Notably, integration of NPs as promising feed supplements for poultry is a way to further improve the overall poultry health and FCR. Until now, there were no data describing the efficacy of G-NHG on broilers performance and its protection against *C. perfringens* infection. The better performance of broiler chickens even after infection with *C. perfringens* could be attributed to the incorporation of garlic extract in the nanoform (nanohydrogel). This is considered a valuable approach used to protect its bioactive ingredients from oxidation controlling its delivery, distribution and storage stability and expanding its shelf life with no effects on the chemical, physical and functional characteristics [26]. The preparation of plant-derived bioactive components by nanotechnology can enhance their activity especially at low dosage [51].

Previous studies described the enhanced effects of garlic active compounds on the digestion of broilers; however, describing this point at the molecular levels has not been investigated until now. Parallel with the increased growth rate and feed efficiency in G-NHG received groups, the expressions of genes encoding digestive enzymes (AMY2A, PNLIP and CCK) were also upregulated. Garlic can stimulate the digestive systems by controlling the digestive pH and the activity of digestive enzymes [52]. Similarly, substances derived from garlic increased the activity of pancreatic enzymes, and thereby, they provided an environment for better nutrient absorption and activated the digestive process [44,53].

The intestinal mucosal barrier mainly consists of epithelial cells, tight junctions between adjacent enterocytes and critical components of the mucosal immune system. The TJP consist of diverse types of proteins, including occludin and JAM-2, which are essential for establishing continuous intact physical barriers between the intestinal epithelial cells [54]. They regulate major immune functions, increase the absorption rate of nutrients, maintain homeostasis and protect against invading pathogens [55]. In the pathogenesis of several inflammatory diseases, disturbances in the production and formation of TJP occur [56]. The disruption of TJP can lead to decreasing nutrient absorption, increasing permeability to the luminal antigens, sustained inflammation, bacteria translocation and tissue damage [57]. Dietary supplementation of essential oils has been documented to strengthen the mucosal barrier and maintain intestinal integrity [19]. In the current study, the expression levels of occludin and *JAM-2* genes were significantly reduced in the infected group, which did not receive G-NHG. However, the expression levels of occludin and *JAM-2* in birds given 400 mg/kg of G-NHG showed 2.06- and 1.8-fold increase when compared to NE-challenged birds fed a basal diet, respectively. This increased expression may be due to improved intestinal barrier functions, especially during the invasion of pathogenic microorganisms. In accordance, supplementation of *Allium hookeri* (AH) root by 3% upregulated the expression of TJP genes; *JAM-2* and occludin in broiler chickens infected by *C. perfringens* [49]. Our results are consistent with previous findings on the active ingredients of other essential oils, where increased TJP gene expression and improved intestinal barrier functions were observed in thymol- and carvacrol-treated broilers challenged with *C. perfringens* [11]. Moreover, Muc-2 is one of the major secreted mucins expressed by intestinal goblet cells and it acts as a protective barrier for the intestine [58,59]. Several studies have demonstrated that necrotic pathogens can induce decreased the expression of *Muc-2* in chickens [60,61]. In agreement with previous studies, decreased expression of *Muc-2* was observed in the NE challenged group compared to the unchallenged group. However, dietary supplementation of higher levels of G-NHG led to upregulation of *Muc-2* expression. Likewise, dietary supplementation of AH at the levels of 1 and 3% dramatically led to increased regulation of *Muc-2* [49]. Moreover, higher mRNA relative expression of *Muc-2* in ileum was found after dietary supplementation of encapsulated essential oils in laying hens [62]. In a recent study, *Muc-2* gene expression was upregulated in broiler chickens challenged with *Salmonella* Typhimurium fed thymol nanoemulsion, indicating the maintenance of the intestinal barrier integrity [16]. Furthermore, nanoencapsulation of cumin essential oil increased *Muc-2* gene expression in broiler chickens [63]. The boosting effects of G-NHG on genes expression of TJP and Muc-2 in the gastrointestinal tract can be attributed to its bioactive compounds, besides its incorporation into the nanoform, which protects and controls its delivery.

Herein, the inclusion of higher levels of G-NHG reduced the serum lipid parameters of broiler chickens even after infection with *C. perfringens*. In accordance, lower triglycerides, total cholesterol, LDL and VLDL levels and higher HDL were detected upon dietary garlic inclusion for broiler chickens when compared with the control group [64]. This can be explained by the possible hypolipidemic and hypocholesterolemic activities of garlic products, which impairs the hepatic functions of cholesterogenic and lipogenic enzymes [65]. Moreover, this effect may be probably due to the inhibition of the Acetyl CoA synthetase enzyme that is necessary for the biosynthesis of fatty acids. In a previous study, garlic extract significantly reduced triglycerides and total cholesterol in diabetic rats [66]. This effect can be explained by the potential antiperoxide action of alliin; the isolated product from garlic or reduction in the hepatic synthesis of VLDL, which considers the precursor of LDL in blood circulation [67].

*Clostridium perfringens* is an important anaerobic pathogen that inhabits the broiler intestine. The proliferation of *C. perfringens* in the poultry intestine results in NE, leading to increased mortality and productivity losses [45]. There are many concerns within the poultry industry to prevent or eliminate the resistance pathogenic bacteria causing gastrointestinal infections [68,69,70,71]. It is well established that garlic extract has been reported to exert an in vitro antimicrobial activity against the potentially pathogenic *C. perfringens* populations [72]. The beneficial effects of garlic supplementation in broiler diets have been previously reported. Nevertheless, there is currently a lack of information about its use in the nanoform as a valuable alternative in diets of broiler chickens to control the pathogenic bacteria. Herein, dietary G-NHG supplementation significantly reduced *C. perfringens* numbers in challenged broiler chickens at 7 and 14 d post challenge (up to 2.837 and 1.857 log_10_ CFU per gram of the ileal digesta, respectively), which in turn alleviated intestinal lesion scores in a dose-related manner. Our present in vivo findings are in accordance with those of early in vitro studies concerning the inhibitory potentials of garlic on C. perfringens. Accordingly, garlic reduced the C. perfringens cecal loads at different supplementation levels compared with the control group [50]. Moreover, Clostridium species counts were significantly decreased in birds supplemented with garlic oil compared with the control group [24]. The antibacterial activity of garlic is mainly attributed to allicin (allyl 2-propenethiosulfinate), which is generated via the enzymatic activity of alliinase on alliin [73]. Interestingly, EO delivery systems, such as nanoemulsions, microcapsules, NPs and liposomes, are models for the enclosure of the natural bioactive compounds to reduce their volatility and improve their effectiveness. Therefore, the tested G-NHG extended the antimicrobial effectiveness and inhibitory spectrum of garlic essential oil in controlling *C. perfringens* by improving the evaluated parameters in broiler chickens. Additionally, garlic essential oil nanoemulsions exhibited a better in vitro antimicrobial activity than the garlic essential oil itself [74].

Indeed, dietary essential oil supplementation has the capability to enhance the broiler chicken’s immune response, which could be involved in affecting the intestinal microbiota and gut health and enhancing the poultry resistance against bacterial infections. To explore the effects of G-NHG on poultry immunity, MPO, NO and lysozyme levels were evaluated. The bactericidal properties of neutrophils and monocytes/macrophages have been attributed to the action of MPO, NO and lysozyme, which are beneficial in terms of the protective immune responses to eliminate the invading bacterial pathogens. Elevated serum MPO, NO and lysozyme activities could be considered to be a response to *C. perfringens* challenge and are vital indicators of inflammatory responses, suggesting activated neutrophils and monocytes in blood [75]. Interfering with MPO, NO and lysozyme production might be helpful for the health status of poultry. Herein, the reducing trend of lysozyme, NO and MPO levels observed in G-NHG-fed birds at 7 and 14 d post challenge demonstrated that dietary G-NHG exerted an important role in mitigating *C. perfringens*-induced stimulation to phagocytes, and subsequently conferring a health benefit to broiler chickens. The enhancing effects exerted on humoral immune response of broiler chickens have been already documented in previous studies post garlic [23,76] and nano garlic [76] supplementation. The positive responses of humoral non-specific defense mechanisms, such as lysozyme activity, and the key mediators of host defense and oxidative tissue injury, such as NO, were reported after garlic extract incorporation into fish feed in a recent study [77]. Moreover, there is very limited information regarding these effects in broilers. To date, the knowledge of the impact of garlic nano materials on the broiler’s immune system has yet to be elucidated. Therefore, more thorough investigations on this subject are still needed. The antibacterial effects of G-NHG on pathogenic *C. perfringens* in the gastrointestinal tract and the stimulatory effects on the immune system may have contributed to the lower mortality rates of broiler chickens in our study, as was previously illustrated [23].

It is interesting to pay attention to the strategy that allows monitoring of the production costs and helps the producer to get the best financial return of the production system. From this point, efforts were made to research the use of phytogenic compounds in the broiler diet with satisfactory effects on the overall performance and economic efficiency [78,79,80]. Under the *C. perfringens* challenge imposed in our research, supplemental 400 mg/kg G-NHG presented a positive impact on the net profit, cost/benefit ratio and total revenue. The profitability ratio and economic efficiency were similar to the NC group, suggesting the use of G-NHG as a potential alternative for the AGPs. In accordance, garlic powder was proved to be the cost effective natural additive [81]. Moreover, supplementation of garlic extract had the highest net return and revenue offering the lowest cost/benefit ratio [82]. Incorporation of garlic extract in the form of nano-oil enhanced its stability and efficiency in improving the production parameters, with great economic benefits.

## 5. Conclusions

Based on our data, it could be concluded that dietary supplementation with G-NHG at 400 mg/kg noticeably enhanced the broilers’ growth performance and maximized the economic returns. Additionally, supplemental G-NHG has a promising role in motivating the birds’ immune response and boosting the expression of genes encoding tight junction protein and mucin-2. This, consequently, counteracted the negative effects of *C. perfringens* challenge through decreasing *C. perfringens* loads, intestinal lesion score and mortality rates. Keeping in view the practical relevance of these findings, G-NHG can be used as a reliable alternative feed additive to the AGPs in the concurrent control or prevention of the economically important enteric diseases.

## Figures and Tables

**Table 1:** Ingredients and chemical composition of the basal diet.

	Starter (1–10 d)	Grower (11–22 d)	Finisher (23–38 d)
Ingredient, %			
Yellow corn	57.9	57.9	63.2
Soybean meal, 48%	35.4	35.4	28.1
Corn gluten, 60%	1.5	1.5	1.5
Soybean oil	1.40	1.4	3.7
Calcium carbonate	0.50	0.5	0.5
Monocalcium phosphate	1.40	1.4	1.2
Common salt	0.30	0.3	0.3
Premix *	0.80	0.8	0.8
DL-Methionine, 98%	0.20	0.20	0.10
Lysine, Hcl, 78%	0.30	0.30	0.20
Antitoxin	0.10	0.10	0.10
Choline chloride	0.20		
Chemical composition			
ME, Kcal/Kg	3003	3106	3200
CP %	23.02	21.50	20.00
EE %	3.88	5.11	6.25
CF %	2.66	2.59	2.52
Ca %	1.18	1.00	0.81
Available P %	0.51	0.47	0.39
Lysine %	1.43	1.26	1.15
Methionine %	0.54	0.50	0.42

* Vitamin and mineral premix per kg of diet: vitamin D3, 5000 IU; vitamin E, 80 IU; vitamin A, 12000 IU; vitamin K3, 3.2 mg; thiamine, 3.2 mg; riboflavin, 8.6 mg; pantothenic acid, 20 mg; folic acid, 2.2 mg; pyridoxine, 4.3 mg; niacin, 65 mg; vitamin B12, 0.15 mg; biotin, 0.20 mg; Fe, 30 mg; Cu, 25 mg; Mn, 115 mg; I, 1.30 mg; Zn, 119 mg and Se, 0.38 mg; ME: metabolizable energy; CP: crude protein; EE: ether extract; CF: crude fiber; Ca: calcium; P: phosphorus.

**Table 2:** Primer sequences of target and reference genes analyzed in a reverse transcription quantitative real-time PCR assay.

Gene	Primer Sequence (5′–3′)	Accession No
*AMY2A*	F-CGGAGTG^↓^GATGTTAACGACTGG R-ATGTTCGCAGACCCAGTCATTG	NM_001001473.2
*PNLIP*	F-GCATCTGGGAAG^↓^GAACTAGGG R- TGAACCACAAGCATAGCCCA	NM_001277382.1
*CCK*	F-AGGTTCCACTGGGAGGTTCT R-CGCCTGCTGTTCTTTAGGAG	XM_015281332.1
occludin	F-ACGGCAAAGCCAACATCTAC R-ATCCGCCACGTTCTTCAC	XM_031604121.1
*JAM-2*	F-AGACAG GAACAGGCAGTGCT R-TCCAATCCCATTTGA GGCTA	XM_031556661.1
*Muc-2*	F-AAACAACGGCCATGTTTCAT R-GTGTGACACTGGTGTGCTGA	NM_001318434
*GAPDH*	F-GGTGGTGCTAAGCGTGTTA R-CCCTCCACAATGCCAA	NM205518

*AMY2A*: alpha 2A amylase; *PNLIP*: pancreatic lipase; *CCK*: cholecystokinin; *JAM-2*: junctional adhesion molecule-2; *Muc-2*: mucin-2; GAPDH: glyceraldehyde 3-phosphate dehydrogenase.

**Table 3:** Effect of feeding different levels of G-NHG on the growth performance of broiler chickens challenged with *C. perfringens.*

	Group	PC	NC	G-NHG, mg/kg				*p* Value	SEM
Parameter				100	200	300	400		
Initial body weight (g/bird)		44.00	44.00	44.20	44.40	44.40	44.60	0.99	0.71
Starter (1–10 d)									
BW, g/bird		320.22 ^d^	321.26 ^c,d^	321.76 ^c,d^	327.08 ^b,c^	331.22 ^b^	339.62 ^a^	<0.001	6.03
BWG, g/bird		275.62 ^d^	277.26 ^c,d^	277.56 ^c,d^	282.68 ^b,c^	287.22 ^b^	295.22 ^a^	<0.001	6.29
FI, g/bird		330.40 ^a^	330.82 ^a^	335.28 ^a^	330.86 ^a^	332.86 ^a^	315.08 ^b^	<0.001	10.33
FCR		1.20 ^b^	1.19 ^b^	1.21 ^b^	1.17 ^b^	1.16 ^b^	1.07 ^a^	<0.001	0.08
Grower (11–22 d)									
BW, g/bird		1208 ^c^	1206 ^c^	1230 ^b^	1245 ^b^	1287 ^a^	1284 ^a^	<0.001	50.20
BWG, g/bird		888 ^c^	885 ^c^	908 ^b^	918 ^b^	955 ^a^	944 ^a^	<0.001	60.26
FI, g/bird		1218 ^a^	1226 ^a^	1226 ^a^	1229 ^a^	1228 ^a^	1167 ^b^	<0.001	18.54
FCR		1.37 ^a^	1.38 ^a^	1.35 ^a^	1.34 ^a^	1.29 ^b^	1.23 ^c^	<0.001	0.07
Finisher (23–38)									
BW, g/bird		1895 ^d^	2402 ^a^	1912 ^d^	2075 ^c^	2261 ^b^	2482 ^a^	<0.001	21.54
BWG, g/bird		686 ^d^	1195 ^a^	682 ^d^	830 ^c^	974 ^b^	1198 ^a^	<0.001	18.63
FI, g/bird		2011 ^c^	2302 ^c^	1968 ^c^	2052 ^b^	2328 ^b^	2556 ^a^	<0.001	29.78
FCR		2.9 ^a^	1.93 ^d^	2.89 ^a^	2.47 ^b^	2.39 ^b^	2.13 ^c^	<0.001	0.05
Overall performance (1–38 d)									
BWG, g/bird		1850 ^e^	2358 ^b^	1868 ^e^	2031 ^d^	2217 ^c^	2438 ^a^	<0.001	18.62
FI, g/bird		3560 ^c^	3859 ^b^	3530 ^c^	3612 ^c^	3889 ^b^	4039 ^a^	<0.001	16.25
FCR		1.92 ^a^	1.63 ^c^	1.89 ^a^	1.78 ^b^	1.75 ^b^	1.66 ^c^	<0.001	0.03

BW: body weight; BWG: body weight gain; FI: feed intake; FCR: feed conversion ratio; PC (positive control): birds fed a basal diet and challenged with *C. perfringens* on d 21, 22 and 23 of the experiment; NC (negative control): birds fed a basal diet; G-NHG (100, 200, 300 or 400 mg/kg)-treated groups: birds fed a basal diet supplemented with 100, 200, 300 or 400 mg/kg of garlic nanohydrogel and challenged with *C. perfringens*; SEM: standard error of the mean. Means with different superscripts within the same row differ significantly (*p* < 0.05).

**Table 4:** Effect of different dietary levels of G-NHG on broiler chickens’ serum immunological parameters at 7 and 14 d post challenge with *C. perfringens.*

Group		At 7 d Post Challenge			At 14 d Post Challenge		
		Lysozyme (U/mL)	MPO (U/L)	NO (μmol/L)	Lysozyme (U/mL)	MPO (U/L)	NO (μmol/L)
PC NC		212.127 ^a^ 186.733 ^c^	32.000 ^a^ 25.300 ^d^	6.383 ^a^ 4.833 ^b^	127.033 ^a^ 72.233 ^f^	29.440 ^a^ 21.033 ^d^	6.383 ^a^ 4.833 ^b^
G-NHG, mg/kg	100	199.417 ^b^	29.067 ^b^	6.033 ^a^	121.430 ^b^	27.450 ^b^	5.240 ^b^
	200	191.137 ^b,c^	29.133 ^b^	5.967 ^a^	101.410 ^c^	26.453 ^b,c^	5.317 ^a,b^
	300	176.823 ^d^	27.513 ^c^	5.240 ^b^	87.373 ^d^	25.407 ^c^	3.940 ^c^
	400	171.000 ^d^	25.427 ^d^	4.243 ^c^	77.817 ^e^	22.127 ^d^	3.210 ^d^
*P* value		<0.001	<0.001	<0.001	<0.001	<0.001	<0.001
SEM		2.148	0.288	0.109	1.077	0.391	0.086

PC (positive control): birds fed a basal diet and challenged with *C. perfringens* on d 21, 22 and 23 of the experiment; NC (negative control): birds fed a basal diet; G-NHG (100, 200, 300 or 400 mg/kg)-treated groups: birds fed a basal diet supplemented with 100, 200, 300 or 400 mg/kg of garlic nanohydrogel and challenged with *C. perfringens*; SEM: standard error of the mean; MPO: myeloperoxidase; NO: nitric oxide. Means with different superscripts within the same row differ significantly (*p* < 0.05).

**Table 5:** Effect of varying dietary G-NHG levels on broiler chicken’s serum biochemical parameters at 38th day of age post challenge with *C. perfringens*.

Group		ALT, U/L	AST, U/L	Uric acid, μmol/L	Creatinine, mg/dL	Cholesterol, mg/dL	Triglycerides, mg/dL	HDL, mg/dL	LDL, mg/dL	VLDL, mg/dL
PC		63.967 ^a^	51.767 ^a^	13.633 ^a^	0.580 ^a^	140.400 ^a^	108.367 ^b^	33.200 ^b^	85.567 ^a^	21.633 ^a^
NC		38.800 ^b^	36.967 ^b^	11.900 ^a,b^	0.403 ^b^	139.760 ^a^	108.167 ^a^	34.333 ^b^	83.753 ^a^	21.673 ^a^
G-NHG, mg/kg	100	39.867 ^b^	34.267 ^b^	10.067 ^b^	0.504 ^a^	136.543 ^a^	101.263 ^bc^	36.680 ^a,b^	79.611 ^a^	20.253 ^a,b^
	200	39.200 ^b^	33.220 ^b^	9.933 ^b^	0.383 ^b^	130.617 ^b^	104.930 ^bc^	37.533 ^a,b^	72.097 ^b^	20.986 ^a,b^
	300	42.900 ^b^	33.233 ^b^	10.367 ^b^	0.411 ^b^	123.300 ^c^	98.263 ^b^	38.067 ^a,b^	65.581 ^b^	19.653 ^b^
	400	43.273 ^b^	33.167 ^b^	11.600 ^a,b^	0.397 ^b^	109.450 ^d^	88.667 ^c^	40.133 ^a^	51.583 ^c^	17.733 ^c^
*p* value		<0.001	<0.001	<0.001	<0.001	<0.001	<0.001	<0.001	<0.001	<0.001
SEM		1.347	1.076	0.664	0.580	1.047	1.767	1.074	1.841	0.353

PC (positive control): birds fed a basal diet and challenged with *C. perfringens* on d 21, 22 and 23 of the experiment; NC (negative control): birds fed a basal diet; G-NHG (100, 200, 300 or 400 mg/kg)-treated groups: birds fed a basal diet supplemented with 100, 200, 300 or 400 mg/kg of garlic nanohydrogel and challenged with *C. perfringens*; SEM: standard error of the mean; ALT: alanine transaminase; AST: aspartate transaminase; HDL: high density lipoprotein; LDL: low density lipoprotein; VLDL: very low-density lipoprotein. Means with different superscripts within the same row differ significantly (*p* < 0.05).

**Table 6:** Economical parameters of broiler chickens fed various levels of G-NHG and challenged with *C. perfringens.*

	Group	PC	NC	G-NHG, mg/kg				SEM	*p* Value
Parameter				100	200	300	400		
Economic parameters ($)									
Feed cost (AVC)		1.5503 ^d^	1.6807 ^c^	1.5941 ^d^	1.6893 ^c^	1.8820 ^b^	1.9774 ^a^	0.011	<0.001
Feed cost/kg body weight		0.8383 ^a^	0.7127 ^b^	0.8535 ^a^	0.8318 ^a^	0.8490 ^a^	0.8419 ^a^	0.007	<0.001
Total expenses		2.7116 ^e^	2.8420 ^c^	2.7554 ^d^	2.8506 ^c^	3.0433 ^b^	3.1387 ^a^	0.011	<0.001
Total revenue		3.4227^d^	4.3393 ^a^	3.4538 ^d^	3.7491 ^c^	4.0838 ^b^	4.3234 ^a^	0.016	<0.001
Net profit		0.7112 ^e^	1.4973 ^a^	0.6984 ^e^	0.8985 ^d^	1.0406 ^c^	1.1847 ^b^	0.022	<0.001
Economic efficiency		0.4594 ^d^	0.8909 ^a^	0.4382 ^d^	0.5321 ^c^	0.5538 ^bc^	0.5991 ^b^	0.016	<0.001
Cost/benefit ratio		1.2625 ^d^	1.5269 ^a^	1.2535 ^d^	1.3153 ^c^	1.3422 ^c^	1.3774 ^b^	0.009	<0.001
Profitability index		0.2075 ^d^	0.3450 ^a^	0.2022 ^d^	0.2396 ^c^	0.2548 ^c^	0.2740 ^b^	0.005	<0.001
Losses due to mortality									
Profit losses		2.2995 ^a^	0.6243 ^b^	0.9980 ^b^	1.1009 ^b^	1.0458 ^b^	0.4973 ^b^	0.266	<0.001
Costs of mortality		8.6910 ^a^	1.1343 ^b^	3.8585 ^b^	3.4253 ^b^	3.0660 ^b^	1.2588 ^b^	0.807	<0.001
Total losses		10.990 ^a^	1.7586 ^b^	4.8564 ^b^	4.5263 ^b^	4.1118 ^b^	1.7561 ^b^	1.05	<0.001
Mortality %		32.00 ^a^	4.00 ^b^	14.00 ^b^	12.00 ^b^	10.00 ^b^	4.00 ^b^	2.76	<0.001

PC (positive control): birds fed a basal diet and challenged with *C. perfringens* on d 21, 22 of the experiment; NC (negative control): birds fed a basal diet; G-NHG (100, 200, 300 or 400 mg/kg)-treated groups: birds fed a basal diet supplemented with 100, 200, 300 or 400 mg/kg of garlic nanohydrogel and challenged with *C. perfringens*; SEM: standard error of the mean. Means with different superscripts within the same row differ significantly (*p* < 0.05).

## Data Availability

The data presented in this study are available on request from the corresponding author.
